# The Maximum Lyapunov Exponent During Walking and Running: Reliability Assessment of Different Marker-Sets

**DOI:** 10.3389/fphys.2018.01101

**Published:** 2018-08-24

**Authors:** Antonis Ekizos, Alessandro Santuz, Arno Schroll, Adamantios Arampatzis

**Affiliations:** ^1^Department of Training and Movement Sciences, Humboldt-Universität zu Berlin, Berlin, Germany; ^2^Berlin School of Movement Science, Humboldt-Universität zu Berlin, Berlin, Germany

**Keywords:** reliability, locomotion, humans, nonlinear dynamics, local dynamic stability, methodology, Lyapunov analysis

## Abstract

The maximum Lyapunov exponent (MLE) has often been suggested as the prominent measure for evaluation of dynamic stability of locomotion in pathological and healthy population. Although the popularity of the MLE has increased in the last years, there is scarce information on the reliability of the method, especially during running. The purpose of the current study was, thus, to examine the reliability of the MLE during both walking and running. Sixteen participants walked and ran on a treadmill completing two measurement blocks (i.e., two trials per day for three consecutive days per block) separated by 2 months on average. Six different marker-sets on the trunk were analyzed. Intraday, interday and between blocks reliability was assessed using the intraclass correlation coefficient (ICC) and the root mean square difference (RMSD). The MLE was on average significantly higher (*p* < 0.001) in running (1.836 ± 0.080) compared to walking (1.386 ± 0.207). All marker-sets showed excellent ICCs (>0.90) during walking and mostly good ICCs (>0.75) during running. The RMSD ranged from 0.023 to 0.047 for walking and from 0.018 to 0.050 for running. The reliability was better when comparing MLE values between blocks (ICCs: 0.965–0.991 and 0.768–0.961; RMSD: 0.023–0.034 and 0.018–0.027 for walking and running respectively), and worse when considering trials of the same day (ICCs: 0.946–0.980 and 0.739–0.844; RMSD: 0.042–0.047 and 0.045–0.050 for walking and running respectively). Further, different marker-sets affect the reliability of the MLE in both walking and running. Our findings provide evidence that the assessment of dynamic stability using the MLE is reliable in both walking and running. More trials spread over more than 1 day should be considered in study designs with increased demands of accuracy independent of the locomotion condition.

## Introduction

Stability is crucial for uninterrupted task execution in dynamic conditions such as locomotion and requires effective regulation by the CNS (Schöner and Kelso, 1988; Massion, 1992; Patla, 2003; Ting et al., 2009; Bohm et al., 2015). As such, dynamic stability during gait refers to the ability of the system to maintain functional locomotion (i.e., not leading to falls) despite the presence of kinematic disturbances or control errors (England and Granata, 2007; Bruijn et al., 2013). One parameter to evaluate numerically the dynamic stability during locomotion is the maximum Lyapunov exponent (MLE) calculated using nonlinear time series analysis and has been adopted as a criterion for the occurrence of control errors (Dingwell and Cusumano, 2000; Buzzi et al., 2003; Bruijn et al., 2013, 2014). The MLE is based on the Lyapunov's theory of dynamic stability, initially formulated to assess the sensitivity of a mechanical system to small perturbations and is often used to quantify how the patterns of gait kinematics change in response to small perturbations (Lyapunov, 1992; Ihlen et al., 2017). While arguments can be made for any of the deriving stability measures, recent reviews suggested the use of the MLE as a prominent measure of dynamic stability (Hamacher et al., 2011; Bruijn et al., 2013; Mehdizadeh, 2017), which has thus received extensive focus in the recent years (Wurdeman et al., 2014; Reynard and Terrier, 2015, 2017; Hamacher et al., 2016; Wu et al., 2016; Chini et al., 2017; Mehdizadeh, 2017; Vieira et al., 2017; Wickstrom et al., 2017).

Although the popularity of the MLE has increased in the context of movement science, there is scarce information on the reliability of the method, especially when comparing measurements performed in a pre-post design after specific therapy or exercise interventions. Previous studies in walking conditions reported good intrasession reliability (Kang and Dingwell, 2006b; van Schooten et al., 2013; Reynard and Terrier, 2014; Reynard et al., 2014; Rábago et al., 2015). Based on this, it was argued that differences between young and older adults (Buzzi et al., 2003; Kang and Dingwell, 2008; Terrier and Reynard, 2015; Mehdizadeh, 2017) as well as patients with moderate neurological gait disorders (Reynard et al., 2014) where instability is expected, can be discovered with the MLE. However, the reliability of the MLE is decreased between days (van Schooten et al., 2013; Reynard and Terrier, 2014). In clinical settings where the evaluation of therapies in a pre-post design is required, the reduced between days reliability provides limitations for the detection of therapy-related alterations. Furthermore, the proof of acute changes after learning or short time adaptation (as for example while walking in different environments), needs a high degree of accuracy (Hak et al., 2012). Based on the reported reliability between days (van Schooten et al., 2013; Reynard and Terrier, 2014), detection of differences after exercise or therapy-induced adaptations might not be feasible. Using a block of measurements within several consecutive days to define a representative value of the MLE might increase the reliability, thus improving the detection ability for small alterations in the MLE. To date there is no information regarding the reliability of the MLE if more measurement days are included in the calculation. Beyond walking, recently several studies investigated the dynamic stability of running using the MLE^37−40^. However, there is no available information regarding intraday or between days reliability of the MLE during running.

Nonlinear time series analysis is a valuable tool for examining the invariants of a dynamical system, but is sensitive to different methodological approaches (Kantz and Schreiber, 2004; Bradley and Kantz, 2015). To date, no consensus exists regarding the data acquisition strategies for the calculation of the MLE. While the computational aspects of the MLE calculation have been frequently examined (Bruijn et al., 2009b; Bradley and Kantz, 2015; Mehdizadeh and Sanjari, 2017; Reynard and Terrier, 2017), there is no comprehensive study examining the placement and clustering regarding data acquisition strategies. Neuromuscular control of the superior segment (trunk) is believed to enable humans maintain stability (Winter et al., 1993) and trunk control to be prioritized over inferior segments (Cromwell et al., 2004). As such, while the trunk is suggested to be representative of the stability of the human system (Kang and Dingwell, 2009; Beurskens et al., 2014), previous studies examining the MLE employed diverse placements and quantities of markers or accelerometers. For instance, the sternum (Terrier and Reynard, 2015), the first (Dingwell and Marin, 2006), and sixth (Bruijn et al., 2009a; van Schooten et al., 2011) thoracic vertebrae, the second (Sejdić et al., 2013) and fifth (Terrier and Dériaz, 2011; van Schooten et al., 2013) lumbar vertebrae have been used, while clusters of two (Wurdeman and Stergiou, 2013) or six markers (Kang and Dingwell, 2006a,b, 2008) have also been employed for acquiring of data and subsequent analysis of dynamic stability. However, through time series analysis we compute a few characteristic numbers from a large sample of data (Kantz and Schreiber, 2004; Bradley and Kantz, 2015), and data collected from different parts of the system can contain different information regarding its states. Possible disparities in the resulting MLE deriving from different bony landmarks, could influence the results of cross-sectional, interventional or prospective study designs and comparisons across different studies that employ the MLE. Moreover, different information regarding the states of the system during locomotion across the different bony landmark positions could have an effect on the resulting reliability. It can be argued that some specific marker sets on the trunk may provide higher reliability than others, representing in a more useful way the dynamic states of the human body during walking and running.

The purpose of the current study was thus to examine the reliability of the MLE both during walking and running using six different marker-sets fixed on the trunk. Further, we aimed to investigate the effects of the different marker-sets on the MLE values. In doing so, we included comparisons of trials performed within the same day, across different days and between block measurements (i.e., three consecutive days of measurement) separated by a long period of time (in average 2 months). We hypothesized dissimilar MLE values within the marker-sets and a marker-set specific reliability during walking and running (i.e., different marker-sets would exhibit different reliability values) and that the reliability would improve in the block design.

## Methods

### Experimental protocol

**Figure 1:**
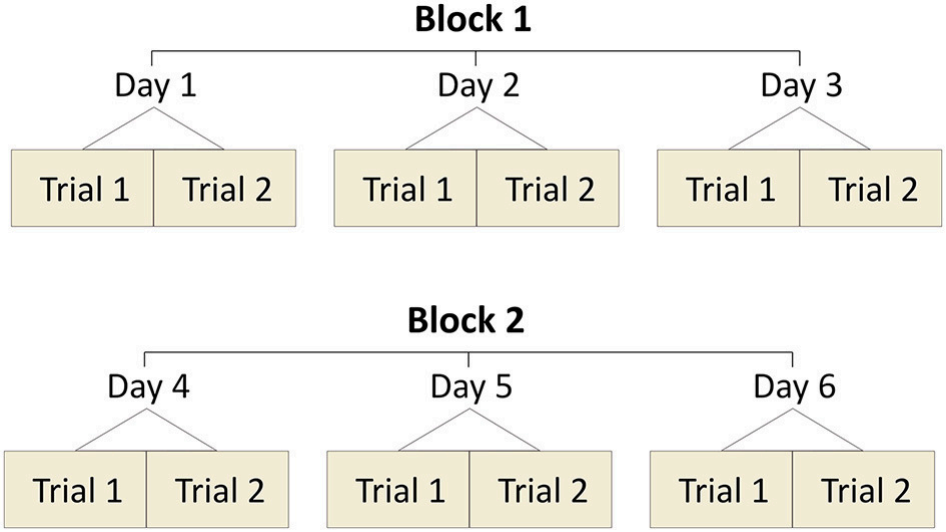
Schematic representation of the measurement design. All participants completed two blocks of measurements. Every block included three consecutive days of measurements (two trials per day). The design was the same in walking and running.

For the current study we recruited 16 young and healthy adults (five female), which were informed of the study's procedures. Anthropometric data of the participants were as follows: 27 ± 5 years of age; 179 ± 9 cm of height; 72 ± 12 kg of body mass. None of the participants had any musculoskeletal or neuromuscular impairments at the time of the measurements or 6 months prior to them. This study was reviewed and approved by the Ethics Committee of the Humboldt-Universität zu Berlin. All the participants gave written informed consent for the experimental procedure, in accordance with the Declaration of Helsinki. The participants came to the lab on six different days in total. Measurements were conducted in two blocks of three consecutive measurement days. The time between blocks was 2 months on average. Each day participants executed randomly (based on computer-generated random numbers) two walking and two running trials at their preferred velocity, separated by a short resting period (60 s). In total, the participants performed 12 walking and 12 running trials per measurement block. The schematic representation of the protocol is depicted in Figure 1. All participants walked and ran on a treadmill (mercury, H-p-cosmos Sports & Medical GmbH, Nussdorf, Germany) with an integrated pressure plate (FDM-THM-S, Zebris Medical GmbH, Germany). A 40-60 s familiarization time preceded each trial. We recorded 270 s for each walking trial and 120 s for each running trial to ensure that a high amount of steps is included in our analysis.

The individuals' preferred velocity was determined while walking and running, through the “method of limits” (Treutwein, 1995). Following a self-selected warm-up, an experienced researcher manipulated the velocity (starting at 0.8 m/s) with varying increments of 0.05–0.08 m/s every 5–10 s. The participant would then affirm when his/her comfort walking velocity was reached and the whole procedure would repeat starting from a higher velocity than the selected. The researcher used similar decrements and the participant once again affirmed his/her preferred pace. The whole process was performed at least two times and until the selected values did not differ more than 10%. The same procedure was followed to determine the preferred running velocity (starting at 1.9 m/s).

### Maximum lyapunov exponents

**Figure 2:**
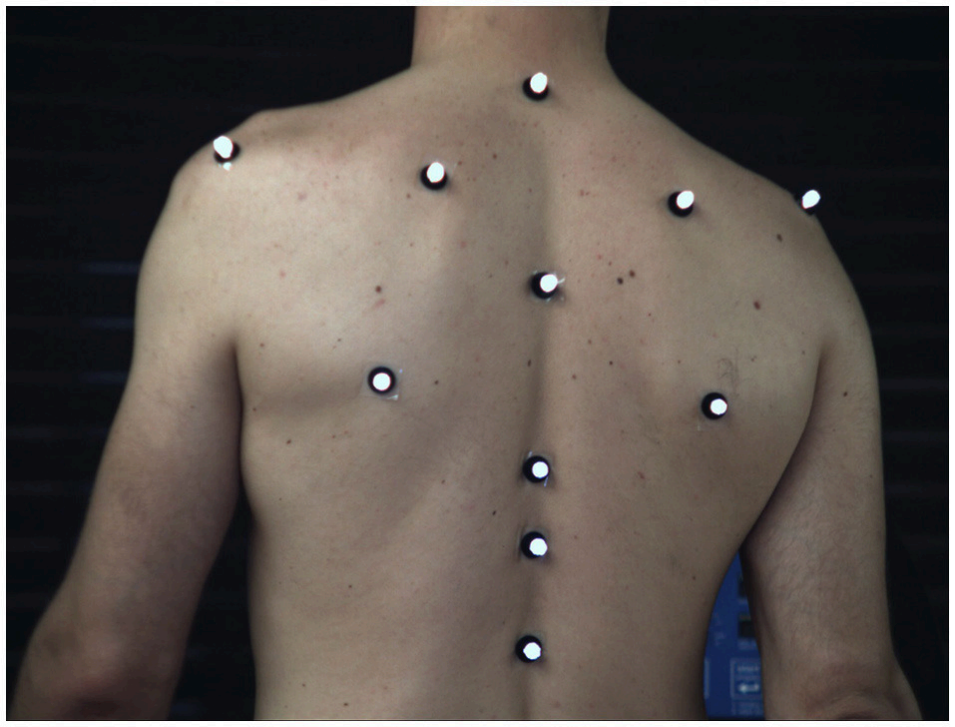
Marker placement on the participants' trunk. Spine: 1st, 6th, 10th, 12th thoracic vertebrae and 2nd lumbar vertebrae. Scapulae: acromion, superior and inferior angle.

Kinematic data were recorded through the use of five high-speed video cameras (Flare 4M180-CCL, IO Industries Inc., Canada) operating at 80 Hz during the walking trials and at 190 Hz during the running trials. We recorded 11 reflective 10 mm-markers positioned on bony landmarks of the trunk. Markers were positioned on the spine at the first (T1), sixth (T6), tenth (T10), and twelfth (T12) thoracic and the second lumbar vertebrae (L2). Further, the scapulae were recorded bilaterally on the acromia, superior and inferior angles (Figure 2). The video tracking was performed using dedicated software (Simi Motion 9.0.4, Simi Reality Motion Systems GmbH, Germany). A fourth order Butterworth 20 Hz low-pass filter was applied to the registered coordinates, maintaining the maximum dynamics of the system (Sinclair et al., 2013). The coordinates of the markers on the T1, T6, T10, and L2 were analyzed separately. Except the time series originating from the individual markers, two clustered marker-sets were created by averaging the coordinates of several markers together on each time frame. The coordinates of all 11 captured markers formed the first clustered marker-set (ALL), while the second marker-set (SP) included only the spine markers (T1, T6, T10, T12, L2) which were clustered together as one.

We calculated the maximum Lyapunov exponents (MLE) on the vertical axis of the six time series, namely the “T1,” “T6,” “T10,” “L2,” “ALL,” “SP.” We analyzed the coordinate data according to the procedure followed in a previous study (Ekizos et al., 2017). In short, we identified the maximum common steps of all participants in all 192 trials (16 participants, 12 trials each) and extracted the data segment corresponding to this amount of steps in each trial. For the walking trials 454 steps were identified in all participants, while in running 279 steps were identified. This segment was then normalized to a uniform data length (based on the recorded steps and the average data points per step). For walking, the data segment consisted of 18614 data points, and for running of 19809 data points.

We reconstructed the state space from the one dimensional time series through delay-coordinate embedding (Packard et al., 1980) as follows:

(1) $$\begin{array}{r} S\left(t\right)=\left[z\left(t\right),z\left(t+\tau \right),\ldots ,z\left(t+\left(m-1\right)\tau \right)\right] \end{array}$$

with S(t) being the *m*-dimensional reconstructed state vector, z(t) the input 1D coordinate series, τ the time delay and *m* the embedding dimension. Time delays were selected based on the first minimum of the Average Mutual Information function (Fraser and Swinney, 1986) and number of embedding dimension through a Global False Nearest Neighbors analysis (Kennel et al., 1992). Individually selected time delays were chosen by averaging the outcome delays of all individual time series for each of the participants (Ekizos et al., 2017). For our data, *m* = 3 was sufficient for all participants in both walking and running, while τ ranged from 12 to 16 in walking (~0.34 of average step) and from 21 to 27 frames (~0.34 of average step) in running. We then calculated the average divergence of each point's trajectory to its closest neighbor, using the Rosenstein algorithm (Rosenstein et al., 1994). The MLE was calculated from the slopes of the resulting average divergence curves' linear fits. The number of data points chosen as the fitting region were equal to one step.

### Statistics

First we performed a repeated measures two-way ANOVA to examine differences in the MLE between the different marker-sets, with trials and marker-sets as within subject factors. If appropriate, *post-hoc* comparisons were made with the Bonferroni correction (adjusted *p*-value for number of comparisons equal to 15) to determine where the effects would be present. Further, a repeated measures three-way ANOVA was employed on the MLE values to test the effect within each day, between days and between blocks separately for the different marker-sets (SPSS v.22, International Business Machines Corp., USA). The two-way mixed single measures absolute agreement intraclass correlation coefficient (ICC) was chosen as more appropriate for our study to determine the reliability of the measurement. To calculate the ICC between trials of same day, trial one against trial two of all days were first assessed and the ICC of all days was subsequently averaged. For the ICC between days the averaged values (trial one and trial two) of all days were used. The ICC was then assessed on the 3 days of block one and block two and the resulting values were averaged. The ICC values between blocks were calculated on the averaged values of all trials in each block. Moreover, to determine the magnitude of the variance in the calculated values of the MLE between the trials of each day, between trials of different days and between all the trials of block one and block two, we calculated the root mean square difference (RMSD). Differences on the absolute MLE values between walking and running were examined through a Student's paired t-test. All statistical tests and procedures were performed separately for the six marker-sets (i.e., four independent markers and two clustered sets) and separately for walking and running. The level of significance for all tests was set to α = 0.05.

## Results

Participants' preferred velocity was 1.5 ± 0.1 m/s in walking and 2.9 ± 0.5 m/s in running. Cadence was 116.3 ± 6.0 steps/min in walking and 160.4 ± 8.7 steps/min in running. The values of the MLE, averaged over all trials, were significantly higher (*p* < 0.001) in running (1.836 ± 0.080) compared to walking (1.386 ± 0.207) in all examined marker-sets, and thus, exhibited that running was locally more unstable than walking.

### Walking reliability

**Figure 3:**
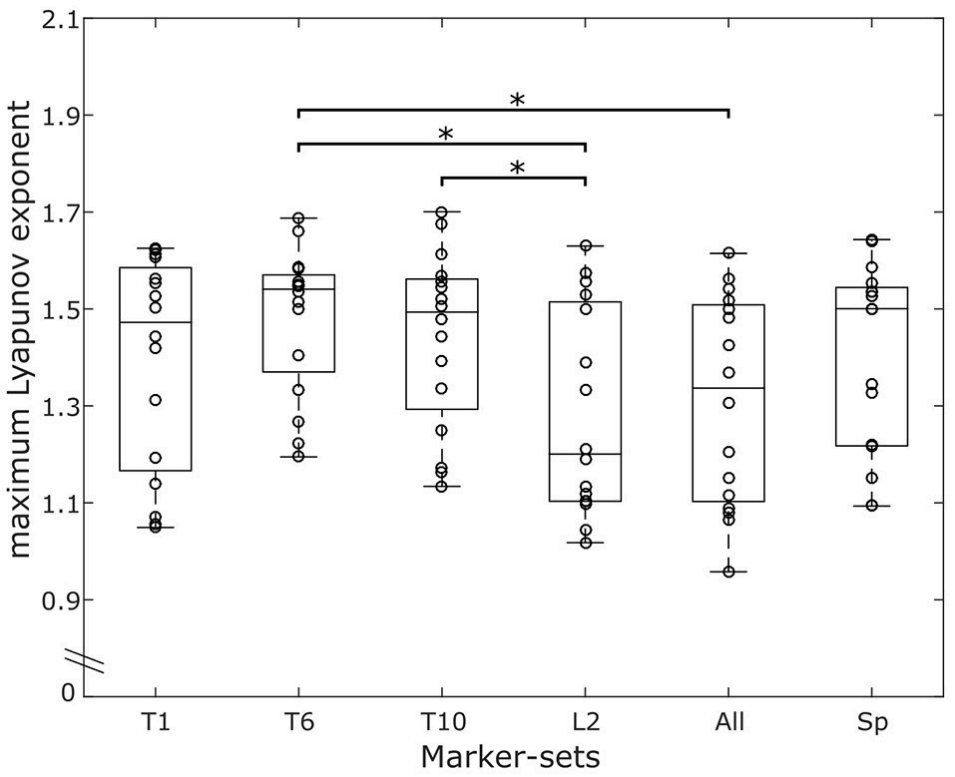
Overlaying graphs of boxplots and scatterplots depicting the maximum Lyapunov exponent (MLE) values in all marker-sets, during walking. Circles exhibit the individual values of the participants. *Statistically significant effect of marker position on the resulting MLE values (*p* < 0.05).

**Figure 4:**
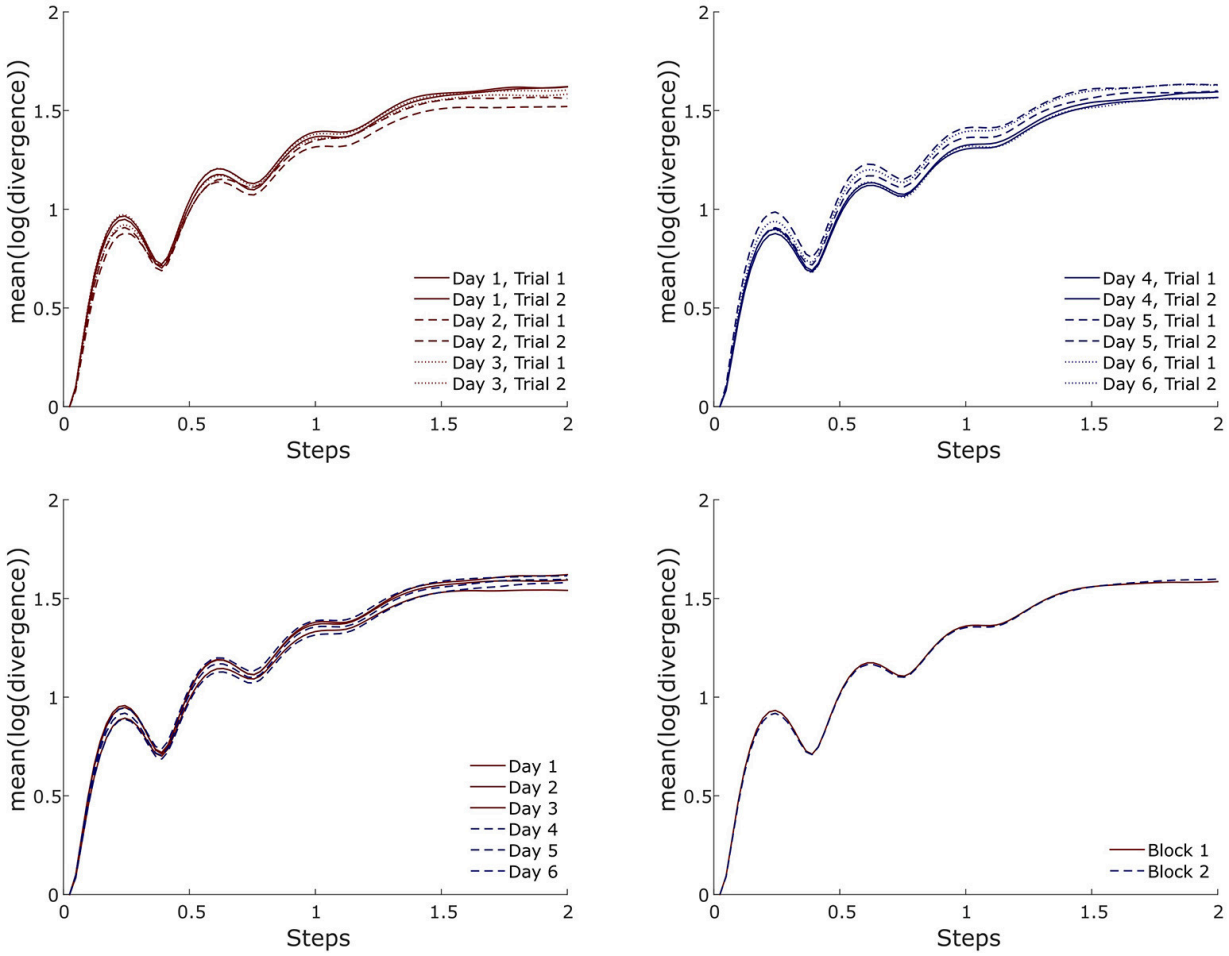
Exemplary (i.e., one participant and one marker-set) divergence curves for all trials, averaged over days and averaged per block during walking.

After the first test on the effect of the marker-sets on the resulting MLE values, we found a statistically significant (*p* < 0.001) effect of the marker-sets. The *post-hoc* comparisons showed significantly higher MLE values on the marker-set “T6” compared to both “L2” (*p* = 0.013) and “ALL” (*p* = 0.021). Moreover, “T10” exhibited significantly higher (*p* = 0.035) MLE values compared to “L2” (Figure 3). For the walking condition, detailed values for the results of the ANOVA, the ICCs and the RMSD of all marker-sets are presented in Table 1. No significant (*p* > 0.05) differences were observed in the MLE in any marker-set when comparing trials of the same day, between consequent days or between blocks. The ICCs for all 6 marker-sets between trials of the same day ranged from 0.946 to 0.980. Between days we observed values of the ICCs ranging from 0.971 to 0.985, while the values of the ICCs between blocks ranged from 0.965 to 0.991. The RMSD exhibited values ranging from 0.042 to 0.047 when considering values of trials within the same days. RMSD values for the between days comparisons (i.e., averaged values of the trials performed in each day) ranged from 0.034 to 0.039. A decrease in the RMSD values was exhibited when considering block values. RMSD values between blocks ranged from 0.023 to 0.034. Although the reliability values were quite high in all marker-sets based on the ICC and RMSD values the marker-set “ALL” exhibited the highest ICC and lowest RMSD within days, between days and between blocks followed by the marker-sets “L2” and “SP.” Both ICCs and RMSDs showed superior values between blocks in all marker-sets compared to the within and between days conditions (Table 1). A similar trend was observed when examining the divergence curves of individual participants for all trials, averaged over days and averaged per block (Figure 4).

**Table 1:** The resulting *p*-values and F statistic from the repeated measures ANOVA, the Intraclass Correlation Coefficient (ICC) with the corresponding upper (U) and lower (L) bounds of confidence intervals and the mean ±standard deviation Root Mean Square Differences (RMSD) for all marker-sets, when considering: both trials within the same day (within days); the averaged values of trials (between days); the averaged values of the consecutive days (between blocks).

**Marker-set**	**Within days**			**Between days**			**Between blocks**		
	**ANOVA**	**ICC**	**RMSD**	**ANOVA**	**ICC**	**RMSD**	**ANOVA**	**ICC**	**RMSD**
T1	*p* = 0.579 *F*_(1, 15)_ = 0.322	0.978	0.045 ± 0.012	*p* = 0.926 *F*_(2, 30)_ = 0.770	0.980	0.039 ± 0.013	*p* = 0.163 *F*_(1, 15)_ = 2.152	0.983	0.033 ± 0.025
		U: 0.992			U: 0.992			U: 0.994	
		L: 0.938			L: 0.954			L: 0.953	
T6	*p* = 0.978 *F*_(1, 15)_ = 0.001	0.946	0.047 ± 0.018	*p* = 0.574 *F*_(2, 30)_ = 0.565	0.971	0.034 ± 0.009	*p* = 0.498 *F*_(1, 15)_ = 0.483	0.965	0.034 ± 0.022
		U: 0.981			U: 0.989			U: 0.988	
		L: 0.858			L: 0.934			L: 0.905	
T10	*p* = 0.911 *F*_(1, 15)_ = 0.013	0.966	0.045 ± 0.018	*p* = 0.531 *F*_(2, 30)_ = 0.646	0.975	0.037 ± 0.010	*p* = 0.292 *F*_(1, 15)_ = 1.192	0.982	0.029 ± 0.020
		U: 0.988			U: 0.991			U: 0.994	
		L: 0.906			L: 0.945			L: 0.951	
L2	*p* = 0.686 *F*_(1, 15)_ = 0.170	0.979	0.042 ± 0.013	*p* = 0.370 *F*_(2, 30)_ = 1.029	0.984	0.036 ± 0.011	*p* = 0.061 *F*_(1, 15)_ = 4.093	0.987	0.027 ± 0.024
		U: 0.993			U: 0.994			U: 0.996	
		L: 0.943			L: 0.963			L: 0.958	
ALL	*p* = 0.924 *F*_(1, 15)_ = 0.009	0.980	0.042 ± 0.013	*p* = 0.244 F_(2, 30)_ = 1.477	0.985	0.034 ± 0.012	*p* = 0.060 *F*_(1, 15)_ = 4.128	0.991	0.023 ± 0.018
		U: 0.993			U: 0.994			U: 0.997	
		L: 0.945			L: 0.967			L: 0.972	
SP	*p* = 0.421 *F*_(1, 15)_ = 0.684	0.973	0.044 ± 0.012	*p* = 0.879 *F*_(2, 30)_ = 0.129	0.982	0.035 ± 0.010	*p* = 0.295 *F*_(1, 15)_ = 1.175	0.984	0.030 ± 0.019
		U: 0.993			U: 0.995			U: 0.996	
		L: 0.945			L: 0.968			L: 0.966	

*The values refer to the walking condition*.

### Running reliability

**Figure 5:**
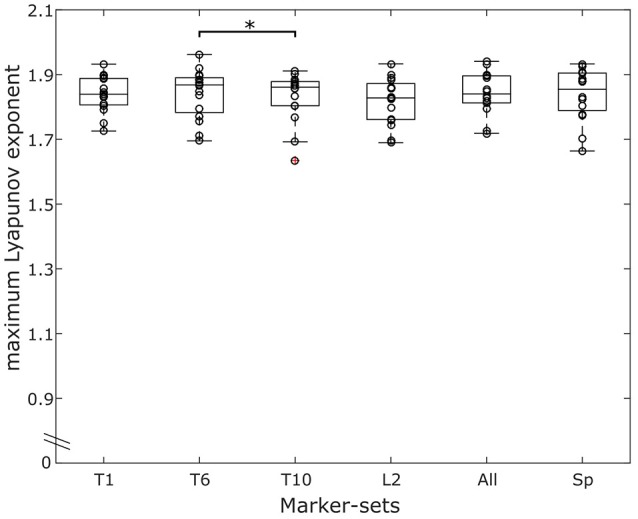
Overlaying graphs of boxplots and scatterplots depicting the maximum Lyapunov exponent (MLE) values in all marker-sets, during running. Circles exhibit the individual values of the participants. *Statistically significant effect of marker position on the resulting MLE values (*p* < 0.05).

**Figure 6:**
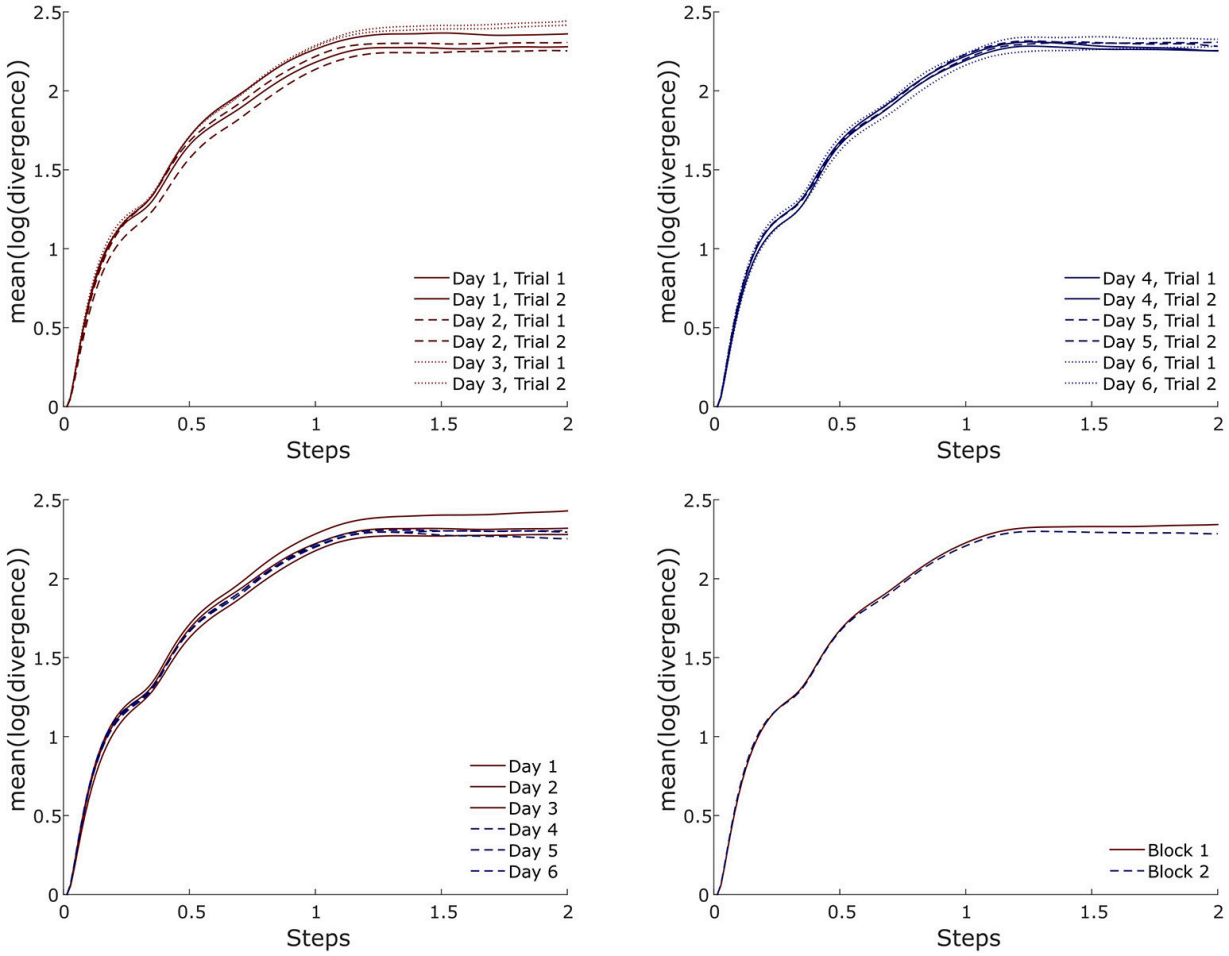
Exemplary (i.e., one participant and one marker-set) divergence curves for all trials, averaged over days and averaged per block during running.

The effect of the marker-sets on the MLE values, was statistically significant (*p* < 0.041). The *post-hoc* comparisons revealed significantly higher MLE values of the marker-set “T6” when compared to the marker-set “L2” (*p* = 0.030) (Figure 5). Similar to walking condition we separately tested each marker-set, and the within days, between days and between blocks effect on the MLE. All values for the results of the repeated measures ANOVA, the ICCs and the RMSD of all marker-sets during the running trials are presented in Table 2. We found significant (*p* = 0.035) differences in the “L2” marker-set when comparing between trials of the same day. No further significant (*p* > 0.05) differences, between trials of the same day, between consequent days or between blocks were found in any other marker-set. ICCs between trials of the same day ranged from 0.739 to 0.844 for all 6 marker-sets, while between days the ICCs ranged from 0.688 to 0.870. Further, the ICC values between blocks ranged from 0.768 to 0.961. RMSD of trials within the same days ranged from 0.045 to 0.050. The RMSD values when considering the between days comparison, ranged from 0.038 to 0.045. Similar to the walking trials a decrease in the RMSD values was found when considering the values of blocks. The RMSD values between blocks ranged from 0.018 to 0.027. During running, the marker-set “SP” exhibited the highest ICC and lowest RMSD between days and between blocks following by the marker-sets “L2” and “T10.” Similar to walking both ICCs and RMSDs showed superior values between blocks in all marker-sets compared to within and between days conditions (Table 2). This was also observed when examining the divergence curves of individual participants for all trials, averaged over days and averaged per block (Figure 6).

**Table 2:** The resulting *p*-values and F statistic from the repeated measures ANOVA, the Intraclass Correlation Coefficient (ICC) with the corresponding upper (U) and lower (L) bounds of confidence intervals and the mean ±standard deviation Root Mean Square Differences (RMSD) for all marker-sets, when considering: both trials within the same day (within days); the averaged values of trials (between days); the averaged values of the consecutive days (between blocks).

**Marker-set**	**Within days**			**Between days**			**Between blocks**		
	**ANOVA**	**ICC**	**RMSD**	**ANOVA**	**ICC**	**RMSD**	**ANOVA**	**ICC**	**RMSD**
**T1**	*p* = 0.516 *F*_(1, 15)_ = 0.442	0.739	0.049 ± 0.014	*p* = 0.431 *F*_(2, 30)_ = 0.865	0.688	0.045 ± 0.017	*p* = 0.825 *F*_(1, 15)_ = 0.050	0.768	0.027 ± 0.031
		U: 0.899			U: 0.864			U: 0.913	
		L: 0.418			L: 0.435			L: 0.449	
**T6**	*p* = 0.055 *F*_(1, 15)_ = 4.319	0.844	0.045 ± 0.012	*p* = 0.718 *F*_(2, 30)_ = 0.334	0.846	0.040 ± 0.016	*p* = 0.130 *F*_(1, 15)_ = 2.565	0.940	0.023 ± 0.014
		U: 0.943			U: 0.937			U: 0.979	
		L: 0.595			L: 0.688			L: 0.835	
**T10**	*p* = 0.116 *F*_(1, 15)_ = 2.782	0.822	0.049 ± 0.013	*p* = 0.932 *F*_(2, 30)_ = 0.071	0.857	0.039 ± 0.013	*p* = 0.073 *F*_(1, 15)_ = 3.729	0.931	0.024 ± 0.017
		U: 0.935			U: 0.942			U: 0.976	
		L: 0.547			L: 0.709			L: 0.802	
**L2**	*p* = 0.035 *F*_(1, 15)_ = 5.341	0.819	0.046 ± 0.015	*p* = 0.738 *F*_(2, 30)_ = 0.307	0.822	0.043 ± 0.011	*p* = 0.231 *F*_(1, 15)_ = 1.559	0.941	0.021 ± 0.015
		U: 0.934			U: 0.927			U: 0.979	
		L: 0.545			L: 0.648			L: 0.842	
**ALL**	*p* = 0.406 *F*_(1, 15)_ = 0.732	0.781	0.050 ± 0.011	*p* = 0.423 *F*_(2, 30)_ = 0.886	0.794	0.042 ± 0.011	*p* = 0.246 *F*_(1, 15)_ = 1.458	0.872	0.027 ± 0.021
		U: 0.918			U: 0.915			U: 0.953	
		L: 0.476			L: 0.599			L: 0.679	
**SP**	*p* = 0.155 *F*_(1, 15)_ = 2.245	0.842	0.048 ± 0.010	*p* = 0.952 *F*_(2, 30)_ = 0.049	0.870	0.038 ± 0.014	*p* = 0.172 *F*_(1, 15)_ = 2.055	0.961	0.018 ± 0.015
		U: 0.943			U: 0.948			U: 0.986	

*The values refer to the running condition*.

## Discussion

In the present study we examined the effect of different marker-sets on the reliability of the MLE computed for each marker-set separately. The study examined these effects on different locomotion conditions, namely walking and running. All marker-sets showed excellent reliability during walking and high reliability in the running condition. The RMSD were lowest when comparing MLE values between blocks, and higher when considering trials of the same day in both walking and running. Further we found that different marker-sets have a significant effect on the MLE values in both walking and running. This effect was more pronounced while walking.

During walking, previous studies in MLE reliability have reported good (i.e., from 0.75 to 0.88) (Portney and Watkins, 2009) intrasession (Kang and Dingwell, 2006b; van Schooten et al., 2013; Reynard and Terrier, 2014; Reynard et al., 2014; Rábago et al., 2015) and moderate (i.e., from 0.53 to 0.68) (Portney and Watkins, 2009) intersession ICC values (van Schooten et al., 2013; Reynard and Terrier, 2014). The ICCs found in our study during walking were clearly higher compared to these previous studies in both intrasession (i.e., from 0.946 to 0.980) and intersession (i.e., from 0.971 to 0.985) comparisons. We recorded kinematic data for 270 s which allowed us to include a high number of steps (i.e., 454 step cycles), and reliability increases substantially as the number of recorded steps increases (Kang and Dingwell, 2006b; Bruijn et al., 2009b). Another source of increased reliability in our study could be the use of the treadmill, in comparison to ambulatory monitoring of gait (van Schooten et al., 2013, 2015). When omitting any averaging between the recorded trials the ICC values between days and between blocks decreased slightly (see Supplementary Material). In walking, all marker-sets were shown to have excellent reliability and provided no significant differences when comparing within days, between days or between blocks. Measuring only one landmark of the trunk during walking could, thus, be sufficient to describe the local dynamic stability of the system and be preferred for reasons of simplification in the study design.

To our knowledge no study has examined reliability on the resulting MLE while running. During running, one marker-set (i.e., L2) showed significant differences in MLE within days and one marker-set (i.e., T1) exhibited consistently low reliability values compared to the others. The clustered marker-set “SP” provided the best and more robust values in the running condition, exhibiting consistently high ICCs and low RMSD within days, across days and between blocks. The improved reliability of the clustered “SP” marker-set may be attributed to small inter-vertebrae movements that are present during locomotion (Syczewska et al., 1999). The inter-vertebrae movements add another layer of complexity to the system and can affect the reliability of the MLE values. By using the clustered marker-set “SP,” these movements would possibly have minimal effects on MLE by repeated measurements due to averaging, thus improving the reliability of the marker-set. It could be suggested that, in studies were a higher measurement reliability is needed for the assessment of the system's local dynamic stability during running, more than one landmark of the trunk should be considered. Conversely, the use of the “T1” marker-set might be less preferable. Moreover, the “SP” marker-set presented no significant differences on the absolute MLE values compared to any of the other marker-sets on the trunk (Figures 3, 5), and thus, the resulting MLE values could also be representative of the dynamic stability of the system. The ICC values during running were slightly lower compared to walking in all marker-sets and the effect of averaging the values of the individual trials more pronounced than in walking (see Supplementary Material). While the motor programming of walking and running remains similar (Cappellini et al., 2006), running exhibits an increased variability and decreased regularity (Estep et al., 2018), which may explain the small decrease of the ICC values. To ensure the differences in walking and running are not dependent on the number of steps, we analyzed our walking data also including 279 steps. When matching the analyzed steps of walking to those recorded in running (i.e., when we analyzed 279 steps in walking), the results in all examined parameters for the reliability and the absolute MLE values remained in similar levels compared to when we included all 454 steps. Our findings confirmed the increased instability during running compared to walking. The higher instability during running may be due to an increased demand in recruiting and coordinating the multiple degrees of freedom faster during the task execution (Jordan et al., 2009; Estep et al., 2018) affecting the assessed within days, between days and between blocks ICCs.

Although reliability was high within and between days, it increased when block measurements were introduced. This can be supported by the results of the reliability analysis with increased ICC and especially by the lower RMSD values. The divergence curves (Figures 4, 6) in both locomotion conditions further evidenced the higher reliability between the blocks. The minimum relative detectable differences (i.e., RMSD divided by the average MLE of the marker-set) were lower in the comparison between blocks. The minimum relative detectable differences results for the “SP” marker-set are 3.1, 2.5, and 2.1% for within days, between days and between blocks in the walking condition and 2.6, 2.1, and 1% respectively for running. It is thus surmised that more trials spread over more than 1 day can significantly improve the reliability of the measurement. To present, studies employing the Lyapunov analysis for examining the stability of gait have focused on differences between groups of young and older age (Buzzi et al., 2003; Kang and Dingwell, 2008; Hamacher et al., 2011, 2015; Terrier and Reynard, 2015; Mehdizadeh, 2017) or health and pathology (Moraiti et al., 2007, 2010; Lamoth et al., 2010; Look et al., 2013; Wurdeman et al., 2013; Kao et al., 2014; Hoogkamer et al., 2015). However, interventional or prospective study designs examining the resulting MLE might require higher degrees of accuracy and thus more than one measurement trial and day.

Based on our results, the chosen marker-set has a significant effect on the absolute value of the MLE on both walking and running conditions and that held true whether the values were obtained from a single or from clustered markers. These differences may be attributed to the nature of the theoretical concept of the used Lyapunov analysis. Time series analysis tries to identify the true dynamics regarding the states of the system from the observed time-ordered data. By measuring in a specific site or local region of the system we approximate the true dynamics, but as with any data collection we gather imperfect information. As such, different components of the system contain different parts of information regarding the states of the system and can yield altered MLE. The absolute MLE values between marker-sets differed up to 13.3% in walking and up to 1.3% in running and therefore highlight the importance of marker placement. Similar to our results, Rispens et al. reported MLE values that differed by 6.7% between two markers on the spine (i.e., when comparing the vertical component of the second and the fifth lumbar vertebrae; Rispens et al., 2014). During walking, MLE typically increases about 8–25% in older compared to young adults (Buzzi et al., 2003; Bruijn et al., 2014; Hamacher et al., 2015). Further, MLE has been reported to increase 9% in patients with focal cerebellar lesion (Hoogkamer et al., 2015) and 21% in patients with various neurological diseases compared to non-affected adults (Reynard et al., 2014), while patients receiving orthopedic shoes exhibited decreased LLE by 9% (Terrier et al., 2013). These values indicate that expected differences can in some cases be affected by different marker-sets or placement errors. During running, changes of 25% have been reported in people with and without lower limb unilateral amputation (Look et al., 2013), which would not be affected by placement differences. However, in milder cases -such as after acute transition from shod to barefoot condition with reported changes of 2% (Ekizos et al., 2017)- the results could be affected from different marker-sets or erroneous marker placement. This indicates that standardization in marker placement and marker-set chosen is important in study designs. Moreover, our findings exhibit the difficulty of comparing the absolute values of MLE between studies, the results of which were obtained with different marker-sets.

## Conclusions

In the current study we endeavored to examine the reliability of the MLE values using different marker-sets within days, across days and between blocks. The chosen marker-set influences the resulting MLE values. The reliability was acceptable in both walking and running for the detection of expected differences in experimental studies. A clustered marker-set may be preferable in the running condition when higher measurement reliability is necessary. More trials spread over more than 1 day, considerably improved the reliability of the MLE measurement and should be considered in study designs with increased demands of accuracy, independent of the locomotion condition.

## Author contributions

AE designed the study, carried out the experiments and data analysis and drafted the manuscript. AlS carried out the experiments and participated in editing the manuscript. ArS participated in data analysis and editing of the manuscript. AA conceived and designed the study and drafted the manuscript. All authors gave final approval for publication.

### Conflict of interest statement

The authors declare that the research was conducted in the absence of any commercial or financial relationships that could be construed as a potential conflict of interest.

## References

[B1] Beurskens R. Wilken J. M. Dingwell J. B. (2014). Dynamic stability of superior vs. inferior body segments in individuals with transtibial amputation walking in destabilizing environments. J. Biomech. 47, 3072–3079. 10.1016/j.jbiomech.2014.06.041 25064425 PMC4163077

[B2] Bohm S. Mademli L. Mersmann F. Arampatzis A. (2015). Predictive and reactive locomotor adaptability in healthy elderly: a systematic review and meta-analysis. Sports Med. 45, 1759–1777. 10.1007/s40279-015-0413-9 26487633 PMC4656697

[B3] Bradley E. Kantz H. (2015). Nonlinear time-series analysis revisited. Chaos 25:097610. 10.1063/1.4917289 26428563

[B4] Bruijn S. M. Meijer O. G. Beek P. J. van Dieën J. H. (2013). Assessing the stability of human locomotion: a review of current measures. J. R. Soc. Interface R. Soc. 10:20120999. 10.1098/rsif.2012.0999 23516062 PMC3645408

[B5] Bruijn S. M. van Dieën J. H. Meijer O. G. Beek P. J. (2009a). Is slow walking more stable? J. Biomech. 42, 1506–1512. 10.1016/j.jbiomech.2009.03.047 19446294

[B6] Bruijn S. M. van Dieën J. H. Meijer O. G. Beek P. J. (2009b). Statistical precision and sensitivity of measures of dynamic gait stability. J. Neurosci. Methods 178, 327–333. 10.1016/j.jneumeth.2008.12.015 19135478

[B7] Bruijn S. M. Van Impe A. Duysens J. Swinnen S. P. (2014). White matter microstructural organization and gait stability in older adults. Front. Aging Neurosci. 6:104. 10.3389/fnagi.2014.00104 24959139 PMC4051125

[B8] Buzzi U. H. Stergiou N. Kurz M. J. Hageman P. A. Heidel J. (2003). Nonlinear dynamics indicates aging affects variability during gait. Clin. Biomech. 18, 435–443. 10.1016/S0268-0033(03)00029-9 12763440

[B9] Cappellini G. Ivanenko Y. P. Poppele R. E. Lacquaniti F. (2006). Motor patterns in human walking and running. J. Neurophysiol. 95, 3426–3437. 10.1152/jn.00081.2006 16554517

[B10] Chini G. Ranavolo A. Draicchio F. Casali C. Conte C. Martino G.. (2017). Local stability of the trunk in patients with degenerative cerebellar ataxia during walking. Cerebellum 16, 26–33. 10.1007/s12311-016-0760-6 26811155

[B11] Cromwell R. Schurter J. Shelton S. Vora S. (2004). Head stabilization strategies in the sagittal plane during locomotor tasks. Physiother. Res. Int. 9, 33–42. 10.1002/pri.298 15132026

[B12] Dingwell J. B. Cusumano J. P. (2000). Nonlinear time series analysis of normal and pathological human walking. Chaos 10, 848–863. 10.1063/1.1324008 12779434

[B13] Dingwell J. B. Marin L. C. (2006). Kinematic variability and local dynamic stability of upper body motions when walking at different speeds. J. Biomech. 39, 444–452. 10.1016/j.jbiomech.2004.12.014 16389084

[B14] Ekizos A. Santuz A. Arampatzis A. (2017). Transition from shod to barefoot alters dynamic stability during running. Gait Posture 56, 31–36. 10.1016/j.gaitpost.2017.04.035 28482203

[B15] England S. A. Granata K. P. (2007). The influence of gait speed on local dynamic stability of walking. Gait Posture 25, 172–178. 10.1016/j.gaitpost.2006.03.003 16621565 PMC1785331

[B16] Estep A. Morrison S. Caswell S. Ambegaonkar J. Cortes N. (2018). Differences in pattern of variability for lower extremity kinematics between walking and running. Gait Posture 60, 111–115. 10.1016/j.gaitpost.2017.11.018 29179051

[B17] Fraser A. M. Swinney H. L. (1986). Independent coordinates for strange attractors from mutual information. Phys. Rev. A 33, 1134–1140. 10.1103/PhysRevA.33.1134 9896728

[B18] Hak L. Houdijk H. Steenbrink F. Mert A. van der Wurff P. Beek P. J.. (2012). Speeding up or slowing down?: gait adaptations to preserve gait stability in response to balance perturbations. Gait Posture 36, 260–264. 10.1016/j.gaitpost.2012.03.005 22464635

[B19] Hamacher D. Hamacher D. Singh N. B. Taylor W. R. Schega L. (2015). Towards the assessment of local dynamic stability of level-grounded walking in an older population. Med. Eng. Phys. 37, 1152–1155. 10.1016/j.medengphy.2015.09.007 26483079

[B20] Hamacher D. Singh N. B. Van Dieën J. H. Heller M. O. Taylor W. R. (2011). Kinematic measures for assessing gait stability in elderly individuals: a systematic review. J. R. Soc. Interface 8, 1682–1698. 10.1098/rsif.2011.0416 21880615 PMC3203491

[B21] Hamacher D. Törpel A. Hamacher D. Schega L. (2016). The effect of physical exhaustion on gait stability in young and older individuals. Gait Posture 48, 137–139. 10.1016/j.gaitpost.2016.05.007 27239774

[B22] Hoogkamer W. Bruijn S. M. Sunaert S. Swinnen S. P. Van Calenbergh F. Duysens J. (2015). Toward new sensitive measures to evaluate gait stability in focal cerebellar lesion patients. Gait Posture 41, 592–596. 10.1016/j.gaitpost.2015.01.004 25618477

[B23] Ihlen E. A. F. van Schooten K. S. Bruijn S. M. Pijnappels M. Dieën V.. (2017). Fractional stability of trunk acceleration dynamics of daily-life walking: toward a unified concept of gait stability. Front. Physiol. 8:516. 10.3389/fphys.2017.00516 28900400 PMC5581839

[B24] Jordan K. Challis J. H. Cusumano J. P. Newell K. M. (2009). Stability and the time-dependent structure of gait variability in walking and running. Hum. Mov. Sci. 28, 113–128. 10.1016/j.humov.2008.09.001 19042050

[B25] Kang H. G. Dingwell J. B. (2006a). A direct comparison of local dynamic stability during unperturbed standing and walking. Exp. Brain Res. 172, 35–48. 10.1007/s00221-005-0224-6 16432700

[B26] Kang H. G. Dingwell J. B. (2006b). Intra-session reliability of local dynamic stability of walking. Gait Posture 24, 386–390. 10.1016/j.gaitpost.2005.11.004 16413784

[B27] Kang H. G. Dingwell J. B. (2008). Effects of walking speed, strength and range of motion on gait stability in healthy older adults. J. Biomech. 41, 2899–2905. 10.1016/j.jbiomech.2008.08.002 18790480 PMC9135052

[B28] Kang H. G. Dingwell J. B. (2009). Dynamic stability of superior vs. inferior segments during walking in young and older adults. Gait Posture 30, 260–263. 10.1016/j.gaitpost.2009.05.003 19502060 PMC3473089

[B29] Kantz H. Schreiber T. (2004). Nonlinear Time Series Analysis. 2nd edn Cambridge, UK ; New York: Cambridge University Press.

[B30] Kao P. C. Dingwell J. B. Higginson J. S. Binder-Macleod S. (2014). Dynamic instability during post-stroke hemiparetic walking. Gait Posture 40, 457–463. 10.1016/j.gaitpost.2014.05.014 24931112 PMC4251664

[B31] Kennel M. Brown R. Abarbanel H. (1992). Determining embedding dimension for phase-space reconstruction using a geometrical construction. Phys. Rev. A 45, 3403–3411. 10.1103/PhysRevA.45.3403 9907388

[B32] Lamoth C. J. Ainsworth E. Polomski W. Houdijk H. (2010). Variability and stability analysis of walking of transfemoral amputees. Med. Eng. Phys. 32, 1009–1014. 10.1016/j.medengphy.2010.07.001 20685147

[B33] Look N. Arellano C. J. Grabowski A. M. McDermott W. J. Kram R. Bradley E. (2013). Dynamic stability of running: the effects of speed and leg amputations on the maximal Lyapunov exponent. Chaos 23:043131. 10.1063/1.4837095 24387570

[B34] Lyapunov A. M. (1992). The general problem of the stability of motion. Int. J. Control 55, 531–534. 10.1080/00207179208934253

[B35] Massion J. (1992). Movement, posture and equilibrium: interaction and coordination. Prog. Neurobiol. 38, 35–56. 10.1016/0301-0082(92)90034-C 1736324

[B36] Mehdizadeh S. (2017). The largest Lyapunov exponent of gait in young and elderly individuals: a systematic review. Gait Posture 60, 241–250. 10.1016/j.gaitpost.2017.12.016 29304432

[B37] Mehdizadeh S. Sanjari M. A. (2017). Effect of noise and filtering on largest Lyapunov exponent of time series associated with human walking. J. Biomech. 64, 236–239. 10.1016/j.jbiomech.2017.09.009 28958634

[B38] Moraiti C. Stergiou N. Ristanis S. Georgoulis A. D. (2007). ACL deficiency affects stride-to-stride variability as measured using nonlinear methodology. Knee Surg. Sports Traumatol. Arthrosc. 15, 1406–1413. 10.1007/s00167-007-0373-1 17828526

[B39] Moraiti C. O. Stergiou N. Vasiliadis H. S. Motsis E. Georgoulis A. (2010). Anterior cruciate ligament reconstruction results in alterations in gait variability. Gait Posture 32, 169–175. 10.1016/j.gaitpost.2010.04.008 20591671

[B40] Packard N. H. Crutchfield J. P. Farmer J. D. Shaw R. S. (1980). Geometry from a Time Series. Phys. Rev. Lett. 45, 712–716. 10.1103/PhysRevLett.45.712

[B41] Patla A. E. (2003). Strategies for dynamic stability during adaptive human locomotion. IEEE Eng. Med. Biol. Mag. 22, 48–52. 10.1109/MEMB.2003.1195695 12733458

[B42] Portney L. G. Watkins M. P. (2009). Foundations of Clinical Research: Applications to Practice. 3rd Edn. Upper Saddle River, NJ: Prentice Hall.

[B43] Rábago C. A. Dingwell J. B. Wilken J. M. (2015). Reliability and minimum detectable change of temporal-spatial, kinematic, and dynamic stability measures during perturbed gait. PLoS ONE 10:e0142083. 10.1371/journal.pone.0142083 26535580 PMC4633040

[B44] Reynard F. Terrier P. (2014). Local dynamic stability of treadmill walking: intrasession and week-to-week repeatability. J. Biomech. 47, 74–80. 10.1016/j.jbiomech.2013.10.011 24200341

[B45] Reynard F. Terrier P. (2015). Role of visual input in the control of dynamic balance: variability and instability of gait in treadmill walking while blindfolded. Exp. Brain Res. 233, 1031–1040. 10.1007/s00221-014-4177-5 25534228

[B46] Reynard F. Terrier P. (2017). Determinants of gait stability while walking on a treadmill: a machine learning approach. J. Biomech. 65, 212–215. 10.1016/j.jbiomech.2017.10.020 29100597

[B47] Reynard F. Vuadens P. Deriaz O. Terrier P. (2014). Could local dynamic stability serve as an early predictor of falls in patients with moderate neurological gait disorders? A reliability and comparison study in healthy individuals and in patients with paresis of the lower extremities. PLoS ONE 9:e100550. 10.1371/journal.pone.0100550 24949737 PMC4065053

[B48] Rispens S. M. Pijnappels M. van Schooten K. S. Beek P. J. Daffertshofer A. van Dieën J. H. (2014). Consistency of gait characteristics as determined from acceleration data collected at different trunk locations. Gait Posture 40, 187–192. 10.1016/j.gaitpost.2014.03.182 24780202

[B49] Rosenstein M. T. Collins J. J. De Luca C. J. (1994). Reconstruction expansion as a geometry-based framework for choosing proper delay times. Phys. Nonlinear Phenom. 73, 82–98. 10.1016/0167-2789(94)90226-7

[B50] Schöner G. Kelso J. A. (1988). Dynamic pattern generation in behavioral and neural systems. Science 239, 1513–1520. 10.1126/science.3281253 3281253

[B51] Sejdić E. Findlay B. Merey C. Chau T. (2013). The effects of listening to music or viewing television on human gait. Comput. Biol. Med. 43, 1497–1501. 10.1016/j.compbiomed.2013.07.019 24034741 PMC4887853

[B52] Sinclair J. Taylor P. J. Hobbs S. J. (2013). Digital filtering of three-dimensional lower extremity kinematics: an assessment. J. Hum. Kinet. 39, 25–36. 10.2478/hukin-2013-0065 24511338 PMC3916920

[B53] Syczewska M. Öberg T. Karlsson D. (1999). Segmental movements of the spine during treadmill walking with normal speed. Clin. Biomech. 14, 384–388. 10.1016/S0268-0033(99)00003-0 10521619

[B54] Terrier P. Dériaz O. (2011). Kinematic variability, fractal dynamics and local dynamic stability of treadmill walking. J. Neuroeng. Rehabil. 8:12. 10.1186/1743-0003-8-12 21345241 PMC3060113

[B55] Terrier P. Luthi F. Dériaz O. (2013). Do orthopaedic shoes improve local dynamic stability of gait? An observational study in patients with chronic foot and ankle injuries. BMC Musculoskelet. Disord. 14:94. 10.1186/1471-2474-14-94 23496924 PMC3608952

[B56] Terrier P. Reynard F. (2015). Effect of age on the variability and stability of gait: a cross-sectional treadmill study in healthy individuals between 20 and 69 years of age. Gait Posture 41, 170–174. 10.1016/j.gaitpost.2014.09.024 25455699

[B57] Ting L. H. van Antwerp K. W. Scrivens J. E. McKay J. L. Welch T. D. J. Bingham J. T.. (2009). Neuromechanical tuning of nonlinear postural control dynamics. Chaos 19:026111. 10.1063/1.3142245 19566271 PMC2832047

[B58] Treutwein B. (1995). Adaptive psychophysical procedures. Vision Res. 35, 2503–2522. 10.1016/0042-6989(95)00016-X 8594817

[B59] van Schooten K. S. Rispens S. M. Elders P. J. Lips P. van Dieën J. H. Pijnappels M. (2015). Assessing physical activity in older adults: required days of trunk accelerometer measurements for reliable estimation. J. Aging Phys. Act. 23, 9–17. 10.1123/japa.2013-0103 24306934

[B60] van Schooten K. S. Rispens S. M. Pijnappels M. Daffertshofer A. van Dieen J. H. (2013). Assessing gait stability: the influence of state space reconstruction on inter- and intra-day reliability of local dynamic stability during over-ground walking. J. Biomech. 46, 137–141. 10.1016/j.jbiomech.2012.10.032 23159098

[B61] van Schooten K. S. Sloot L. H. Bruijn S. M. Kingma H. Meijer O. G. Pijnappels M.. (2011). Sensitivity of trunk variability and stability measures to balance impairments induced by galvanic vestibular stimulation during gait. Gait Posture 33, 656–660. 10.1016/j.gaitpost.2011.02.017 21435878

[B62] Vieira M. F. Rodrigues F. B. de SSá. E Souza G. S. Magnani R. M. Lehnen G. C. Andrade A. O. (2017). Linear and nonlinear gait features in older adults walking on inclined surfaces at different speeds. Ann. Biomed. Eng. 45, 1560–1571. 10.1007/s10439-017-1820-x 28293751

[B63] Wickstrom J. Stergiou N. Kyvelidou A. (2017). Reliability of center of pressure measures for assessing the development of sitting postural control through the stages of sitting. Gait Posture 56, 8–13. 10.1016/j.gaitpost.2017.04.031 28477560 PMC5511624

[B64] Winter D. A. MacKinnon C. D. Ruder G. K. Wieman C. (1993). An integrated EMG/biomechanical model of upper body balance and posture during human gait. Prog. Brain Res. 97, 359–367. 8234761 10.1016/s0079-6123(08)62295-5

[B65] Wu Y. Li Y. Liu A. M. Xiao F. Wang Y. Z. Hu F.. (2016). Effect of active arm swing to local dynamic stability during walking. Hum. Mov. Sci. 45, 102–109. 10.1016/j.humov.2015.10.005 26615477

[B66] Wurdeman S. R. Myers S. A. Jacobsen A. L. Stergiou N. (2014). Adaptation and prosthesis effects on stride-to-stride fluctuations in amputee gait. PLoS ONE 9:e100125. 10.1371/journal.pone.0100125 24956384 PMC4067312

[B67] Wurdeman S. R. Myers S. A. Stergiou N. (2013). Transtibial amputee joint motion has increased attractor divergence during walking compared to non-amputee gait. Ann. Biomed. Eng. 41, 806–813. 10.1007/s10439-012-0705-2 23180032 PMC3596479

[B68] Wurdeman S. R. Stergiou N. (2013). Temporal structure of variability reveals similar control mechanisms during lateral stepping and forward walking. Gait Posture 38, 73–78. 10.1016/j.gaitpost.2012.10.017 23245640

