# Supplementary material for: The Maximum Lyapunov Exponent During Walking and Running: Reliability Assessment of Different Marker-Sets
Source: Front Physiol. 2018 Aug 24;9:1101. doi: 10.3389/fphys.2018.01101 (PMC6117405; doi:10.3389/fphys.2018.01101)
Supplement: Supplementary file 1 [file Table_1.docx]

Supplementary Material

The maximum Lyapunov exponent during walking and running: reliability assessment of different marker-sets

Antonis Ekizos^1,2^, Alessandro Santuz^1,2^, Arno Schroll^1,2^, Adamantios Arampatzis^1,2*^

*** Correspondence:** Adamantios Arampatzis: a.arampatzis@hu-berlin.de

# Supplementary Table

Table 1. The resulting ICC values when trials were not averaged and the values corresponding to each trial were used (i.e. the six trials in each block were compared to each other for the between days ICC and all twelve trials were considered for the between blocks ICC).

| **Marker-set** | **Between days ICC** | | **Between blocks ICC** | |
| --- | --- | --- | --- | --- |
|  | Walking | Running | Walking | Running |
| **T1** | 0.857 | 0.629 | 0.965 | 0.639 |
| **T6** | 0.894 | 0.793 | 0.933 | 0.789 |
| **T10** | 0.903 | 0.789 | 0.955 | 0.752 |
| **L2** | 0.904 | 0.764 | 0.970 | 0.737 |
| **ALL** | 0.891 | 0.721 | 0.974 | 0.665 |
| **SP** | 0.921 | 0.809 | 0.973 | 0.799 |
